# Single-Stage Revision Reverse Shoulder Arthroplasty: Preoperative Planning, Surgical Technique, and Mixed Reality Execution

**DOI:** 10.3390/jcm11247422

**Published:** 2022-12-14

**Authors:** Kristine Italia, Marine Launay, Luke Gilliland, James Nielsen, Roberto Pareyon, Freek Hollman, Asma Salhi, Jashint Maharaj, Mohammad Jomaa, Kenneth Cutbush, Ashish Gupta

**Affiliations:** 1 Queensland Unit for Advanced Shoulder Research (QUASR), Queensland University of Technology, Brisbane, QLD 4000, Australia; 2 Akunah, Brisbane, QLD 4120, Australia; 3 Greenslopes Private Hospital, Brisbane, QLD 4120, Australia; 4 Brisbane Private Hospital, Brisbane, QLD 4000, Australia

**Keywords:** single-stage revision, shoulder arthroplasty, preoperative planning, mixed reality

## Abstract

Revision shoulder arthroplasty is increasing with the number of primary shoulder replacements rising globally. Complex primary and revisions of shoulder arthroplasties pose specific challenges for the surgeon, which must be addressed preoperatively and intraoperatively. This article aimed to present strategies for the management of revision of shoulder arthroplasties through a single-stage approach. Preoperatively, patient factors, such as age, comorbidities, and bone quality, should be considered. The use of planning software can aid in accurately evaluating implants in situ and predict bony anatomy that will remain after explantation during the revision surgery. The planning from such software can then be executed with the help of mixed reality technology to allow accurate implant placement. Single-stage revision is performed in two steps (debridement as first step, implantation and reconstruction as the second step), guided by the following principles: adequate debridement while preserving key soft tissue attachments (i.e., rotator cuff, pectoralis major, latissimus dorsi, deltoid), restoration of glenoid joint line using bone grafting, restoration of humeral length, reconstruction and/or reattachment of soft tissues, and strict compliance with the postoperative antibiotic regimen. Preliminary results of single-stage revision shoulder arthroplasty show improvement in patient outcomes (mean 1 year), successful treatment of infection for those diagnosed with periprosthetic joint infection, and improved cost–benefit parameters for the healthcare system.

## 1. Introduction

As the number of shoulder joint replacements increases globally, the number of revision procedures is predicted to increase greatly. The Australian Orthopaedic Association National Joint Replacement Registry (AOANJRR) comprehensively captured this trend and reported an increase of 220% in the number of primary shoulder replacements since 2008, with the proportion of total shoulder replacements (anatomic and reverse shoulder replacements) increasing from 58% in 2008 to nearly 90% in 2021 [1]. This has resulted in a higher number of associated revision shoulder arthroplasties. In 2021, 7.3% of all shoulder replacements were revision procedures. However, a further classification of revision procedures can be identified based on the type of revision executed. The Australian Registry reported that 54% of the revisions of total stemmed arthroplasties occurred because of the humeral component, and 28% of this group occurred in a complex case whereby both the primary humeral and glenoid components were revised [1]. In addition, further analysis showed that the cumulative percentage of cases with a re-revision of a primary revision was 24% in Reverse Shoulder Arthroplasty (RSA) and 20% in non-RSA at 8 years [2].

Instability-related complications are the second most dominant factor for revision surgery in the setting of stemmed total shoulder arthroplasty (TSA), accounting for 27% of the revision cases [1]. It is the number one reason for revision after RSA, with nearly 33% of the cases [1]. Anatomic TSA failure rates are predominantly associated with rotator cuff failure, instability, glenoid loosening, and infection [1,3,4]. In RSA, the top causes of failure include humeral-associated complications, predominantly in the form of loosening and proximal humeral stress shielding, instability/dislocation, and infection [1,3,5,6,7,8,9,10]. Nerve-related complications account for a minority of revisions in the case of reverse shoulder replacement procedures [5,11].

Revisions to treat underlying infection remain the dominant reason for revision shoulder arthroplasty. Traditionally most surgeons would perform a two- or three-stage revision procedure in the setting of an infection. This has resulted in modest outcomes at best. Data from the hip and knee literature demonstrate that a single-stage reconstructive procedure for arthroplasty has resulted in satisfactory outcomes [12,13,14,15]. While there is a lack of literature regarding single-stage shoulder revisions for the treatment of infection [16,17,18], the senior author in this paper has been performing single-stage revisions routinely for all arthroplasties, be them infected or not.

In the revision setting, it is important to know the positioning/malpositioning of the primary implant in situ and the available glenoid and humeral bone stock. This understanding of the pathological joint will allow for the correct positioning of the revised implants. Failure to do so could result in further revisions due to instability, insufficient glenoid stability, and humerus-related complications.

In this article, we shall address how preoperative planning with metal artifact reduction for revision shoulder surgery is paramount to achieving accurate reconstructions, aiding surgeons in ascertaining the implant position preoperatively, and thereby promoting single-stage reconstructive procedures to achieve better clinical outcomes. We will also discuss the potential of intraoperative use of Mixed Reality (MR) technology as an execution tool to assist in bridging the gap between preoperative planning and surgical execution.

## 2. Preoperative Planning for Revision Shoulder Arthroplasty

For satisfactory revision arthroplasty to be embarked upon, several factors must be evaluated and addressed individually. These include patient factors, glenoid and humerus pathology, their premorbid morphology, soft tissue envelope and associated soft tissue reconstruction, existing implants in situ, and revision implants to be considered (Table 1). We shall address these factors and how preoperative planning and mixed reality technology will aid in evaluating and executing complex revision shoulder arthroplasty procedures.

### 2.1. Patient Factors

A variety of patient factors need to be evaluated and addressed before embarking on a single-stage revision arthroplasty. These generally relate to the patient/host’s ability to have good bone and soft tissue healing. Secondly, in diagnosed periprosthetic joint infection (PJI), the organism and its virulence play a dominant role [19]. Good patient selection and optimization are of utmost importance for excellent outcomes postoperatively.

Age at the time of surgery is critical. Like any other major surgical procedure, elderly patients are more fragile and at higher risk of complications. Medical comorbidities play a major role as well. The number of medical comorbidities classifies patients into type A (uncompromised), B (with one to two compromising factors), and C (more than two compromising factors), with type C having more compromising systemic factors that can impair immune system response to infection and single-stage revision [20]. Lum et al. stated that an increase in the number and severity of patient’s comorbidities could result in higher rates of complications and even death [19]. Systemic factors such as diabetes, smoking, malnutrition, and BMI need to be properly controlled prior to single-stage revision surgery, as these play a role intraoperatively, in the length of hospital stay, and in the postoperative recovery of patients. PJI is one of the reasons for revision surgery. If blood sugar is poorly controlled, defined as having uninterrupted glycosylated hemoglobin of more than 8.0% for 1 year or more despite standard care [21], infection control will also be challenging, which may make single-stage revision unsuccessful. Smoking is known to affect lung function and wound healing. Hence, patients are encouraged to discontinue smoking preoperatively to optimize them for surgery, avoid the possible anesthetic complications related to poor lung function, and promote better surgical site healing. The use of anticoagulants is also noted preoperatively, as this will increase the risk of bleeding intraoperatively and the risk of hematoma formation postoperatively, which subsequently affects wound healing and increases the risk of postoperative infection [22]. Aside from these systemic factors, it is also ideal to evaluate the patient’s bone density as this provides information on bone quality, which can affect the revision surgery. It has been shown that a diagnosis of osteoporosis increases the risk of periprosthetic fracture and revision shoulder arthroplasty [23]. Often, the decision for implant retention is purely related to the medical comorbidity of the patient rather than surgical desire. A relatively simple, efficient, and quick surgical revision may be contemplated in frail patients.

### 2.2. Implants and Bony Anatomy

#### 2.2.1. Existing Implants

Evaluation of the primary implants in situ is one of the first steps to be undertaken. Often the primary implants can be left in place, and a complete reconstruction or revision can be avoided. One of the critical factors indicating implant retention is to evaluate the primary implants’ position.

**Figure 1:**
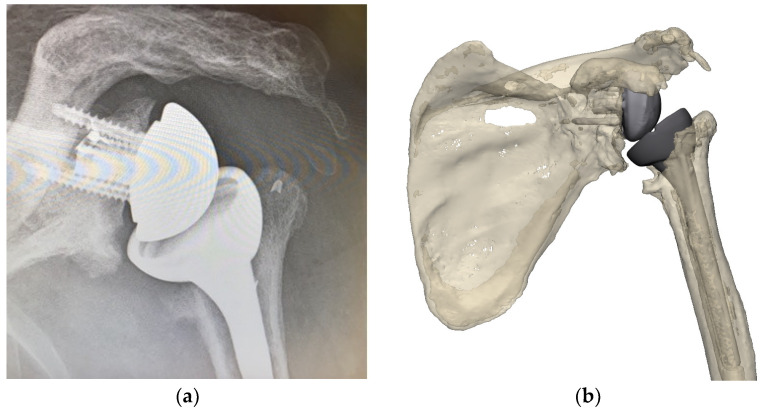
Preoperative X-ray of glenohumeral joint (**a**) and 3D reconstruction showing malpositioning of primary implants in situ (image from Reflect COMPLEX^TM^ (Akunah, Brisbane, Australia)) (**b**).

The use of preoperative planning with advanced three-dimensional (3D) reconstruction techniques allows the surgeon to evaluate the degree of potential implant malposition and whether this can be compensated (Figure 1). In cases where malposition cannot be compensated for, strategies must be employed to remove the existing implants and proceed with the new implantation.

#### 2.2.2. Bony Anatomy

Once the existing implant factors have been assessed, and complete removal and replacement of the implants in situ are necessary, the assessment of the bony anatomy and resultant morphological deformities is critical as part of the preoperative planning process.

**Figure 2:**
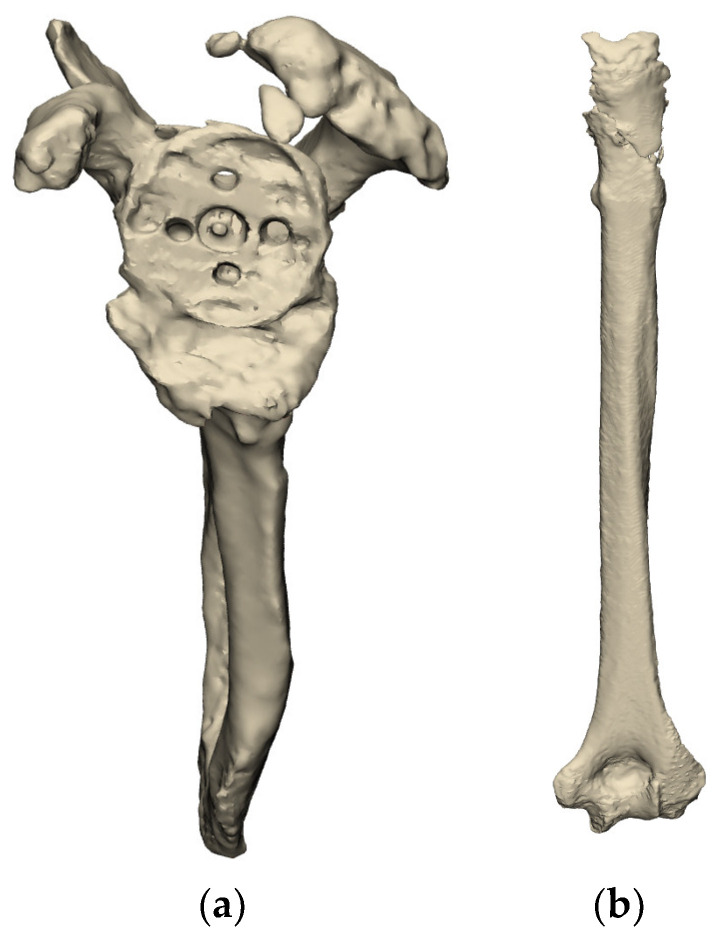
3D reconstructions of the scapula (**a**) and humerus (**b**) after subtraction of the primary implants in situ (images from Reflect COMPLEX^TM^ (Akunah, Brisbane, Australia)).

Use of complex metal artifact reduction algorithms can be used to subtract the primary implants, thus allowing for the assessment of the underlying glenoid and/or humerus deformity, as shown in Figure 2. The senior author has been using the REFLECT COMPLEX^TM^ Revision Planning Service from Akunah, Brisbane, Australia, which will be referenced throughout this paper. This planning software provides a solution to address metal artifacts, which is the main impediment when planning single-stage revision shoulder arthroplasties. It has the capacity to perform metallic implant subtraction and subsequently segment preoperative computed tomography (CT) scans to produce 3D models of the scapula and the humerus. This then allows surgeons to evaluate the remaining bone stock and accurately plan for single-stage surgery.

##### Glenoid

*Evaluation of the Glenoid Bone Loss*

Complex primary and revision arthroplasty may result in complex 3D glenoid bone defects [24,25]. The evaluation of these defects in a 3D pattern must be performed holistically as part of the preoperative planning process. The Gupta-Seebauer classification allows for a 3D quantification of glenoid bone defects based on the centricity or eccentricity of the defect, and guides their reconstruction with a proposed treatment algorithm [26]. It also defines the so-called “50% rule”, which must be employed later when planning new implant types and positioning [26].

*Residual Bone Stock available for Reconstruction*

**Figure 3:**
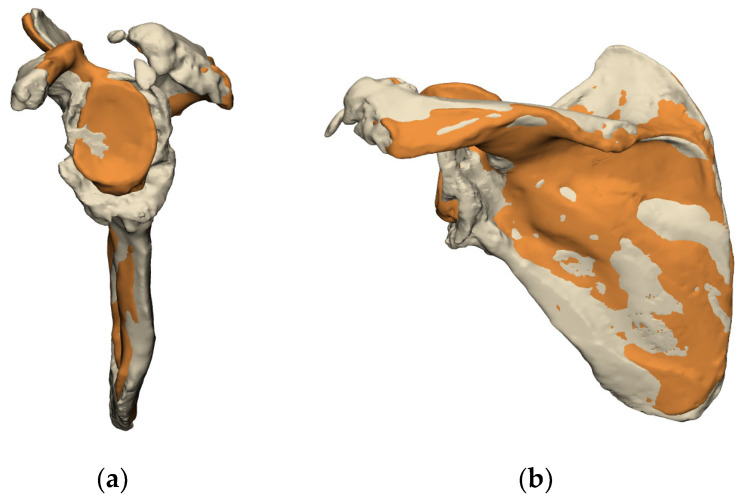
Use of overlaying techniques to assess reconstruction techniques; glenoid enface view (**a**) and posterior view (**b**) (images from Reflect COMPLEX^TM^ (Akunah, Brisbane, Australia)).

Once the amount of bone loss is evaluated, the use of overlaying techniques can be employed to further assess the amount of residual bone stock available and the type of reconstruction required (Figure 3).

Statistical Shape Modelling (SSM) techniques can be used to predict a patient’s premorbid anatomy from the pathological anatomy. Using a scapular SSM allows a personalized prediction of the premorbid glenoid anatomy calculated from the pathological one [27]. This provides the surgeon with valuable information about the premorbid anatomy of the glenoid and scapula and assists the surgeon in three ways:

1. Evaluation of the volumetric bone loss in the glenoid,
2. Development of a framework for reconstruction modalities: use of bone grafts, metal augments, or custom implants.
3. Most critically, it enables the surgeon to restore the glenoid joint line.

*Glenoid Joint Line Concept*

**Figure 4:**
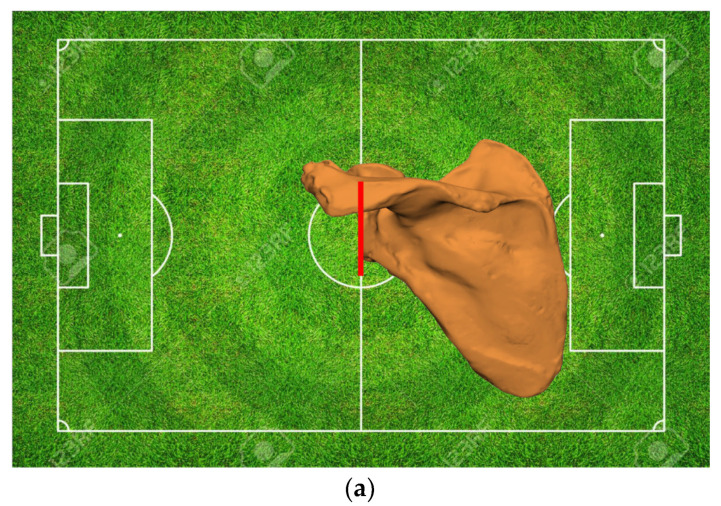

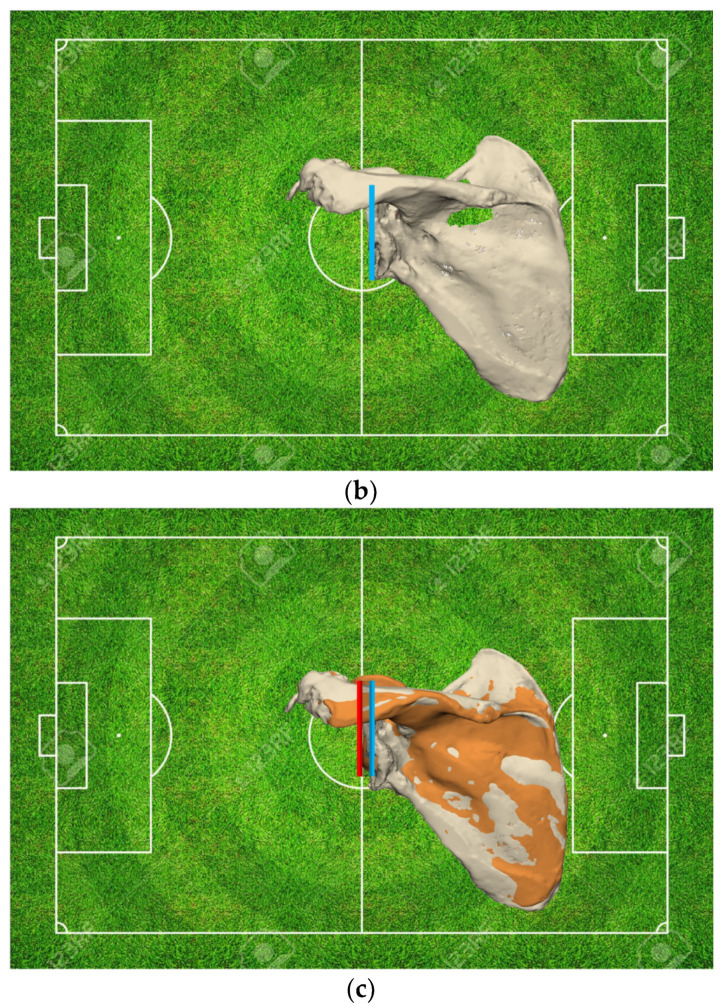
Evaluation of the premorbid anatomy allows to locate the native joint line (red line) (**a**) and the subsequent amount of medialization in the pathological anatomy (blue line) (**b**), thus providing information on the reconstruction necessary to restore the native joint line (distance between the blue and red lines) (**c**).

Each pathological glenoid is the result of some degree of bone wear or defect, thereby medializing the native glenoid joint line. Numerous studies have demonstrated that the glenoid joint line sits approximately 8 to 10 mm from the base of the coracoid [28] or around 27 to 29 mm from the scapular notch for females and males, respectively [29]. These morphological relationships, paired with 3D reconstructions of the anatomy and a scapular SSM, can be used to estimate the premorbid joint line. This information assists the surgeon in ascertaining the severity of the defect and the required amount of lateralization or medialization necessary for the restoration of the native glenoid joint line, thereby guiding the surgeon toward restoring the glenohumeral and scapulothoracic kinematics, as well as assisting in soft tissue reconstruction (Figure 4).

In addition, knowledge of the premorbid glenohumeral joint line during reconstructive procedures allows for an adequate length-tensioning relationship of the rotator cuff, the conjoint tendon, and the deltoid, as well as the periscapular muscles.

Restoration of the glenoid joint line is one of the cornerstones of revision shoulder arthroplasty, as the foundation of the revision implant lies in the satisfactory primary stability and positioning of the glenoid baseplate [26]. Glenoid baseplate malpositioning and/or inadequate primary fixation are associated with modest outcomes and reduced implant longevity [30].

##### Humerus

**Figure 5:**
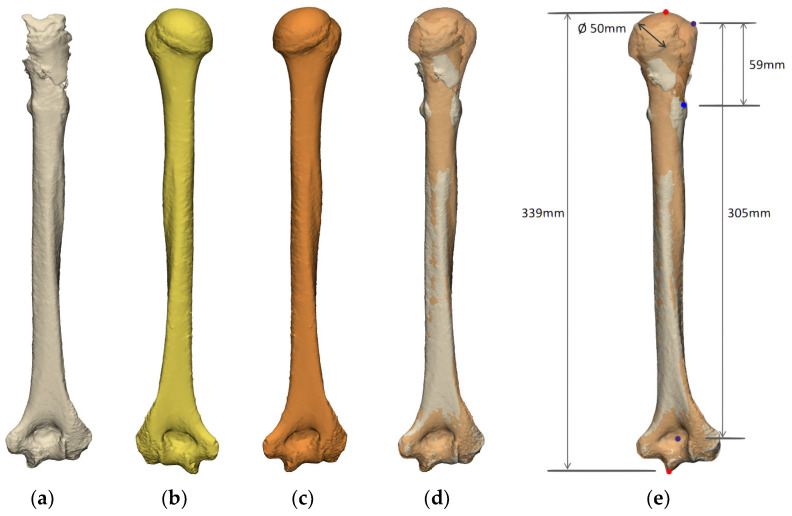
Evaluation of the humerus native anatomy (**a**) combined with the use of the contralateral side (**b**), which is mirrored (**c**) and overlaid onto the native anatomy (**d**) to ascertain the humeral length and subsequent amount of humeral bone loss (**e**) (images from Reflect COMPLEX^TM^ (Akunah, Brisbane, Australia)).

Glenohumeral joint instability is the second cause of revision shoulder arthroplasty, which is predominantly linked to implant malposition and associated impingement [1]. Other significant factors for this are the failure to ascertain the humeral length and the failure to restore humeral anatomy [5,31,32]. Standardized preoperative planning for revision arthroplasty should involve evaluating the proximal humeral deficiency (Figure 5). This highlights the importance of imaging the full humerus, including the epicondyles, to ensure an accurate referencing system to establish the full length of the humerus and its subsequent potential deficiency. Traditionally, radiographs of the contralateral arm are collected to measure humeral length. However, the contralateral shoulder may also be pathologic, or arthroplasty may be present on the contralateral side. When adequate size relationships cannot be established, SSM of the humerus could be used to calculate and evaluate the proximal humeral bone deficiency.

#### 2.2.3. Planning of Implants

Implant positioning in the revision setting is one of the biggest challenges faced in arthroplasty [33,34,35,36]. An agnostic planning and templating software is important to be able to address the multi-factorial challenge revision arthroplasties can be. Often in a revision setting, the surgeon is faced with a restricted choice of implants which might not be their preferred option. The ability to choose reconstructive implants based on generic designs and dimensions, rather than implant type and sizes from only one manufacturer, empowers surgeons to tackle each revision more astutely. In addition, preoperative planning of revisions should account for the ability to mix and match implants from different manufacturers depending on the patient’s need.

##### Glenoid

Once glenoid morphological deformities are assessed and the joint line restoration is calculated, the surgeon can then use the revision planning software to position the implants for the needs of each patient. Implant positioning is based on a host of patient-related and implant-related factors, the choice of implants used by the surgeon training, and availability of devices. The “50% rule” described by Seebauer et al. recommends 50% of the glenoid peg and that two locked screws must be fixed in the native scapula. In addition, a minimum of 50% of the baseplate-bone graft construct must be in contact with the native scapula for primary implant stability [26].

**Figure 6:**
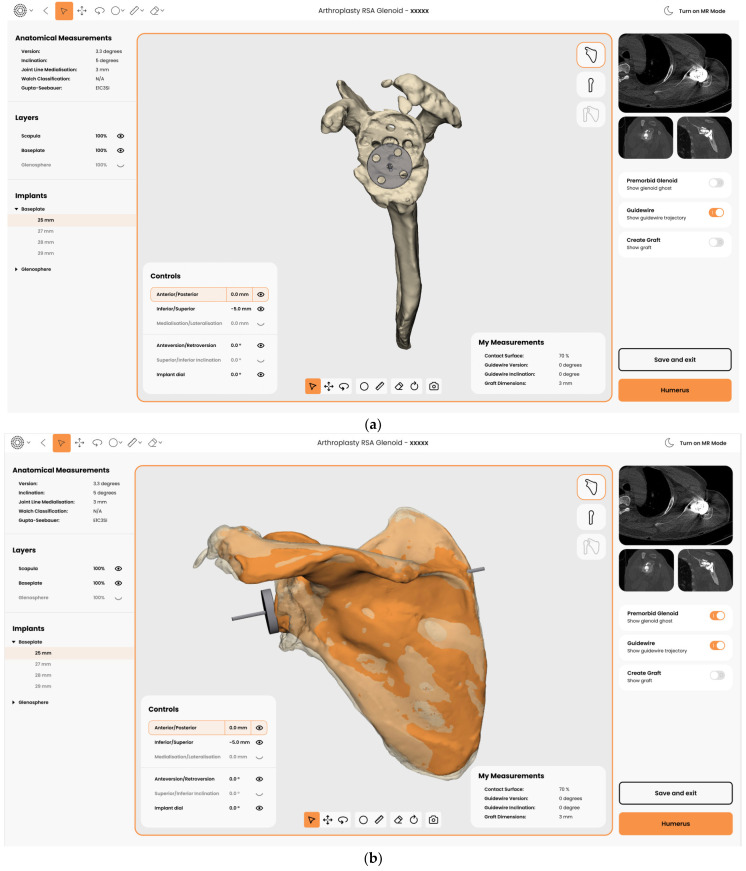
Planning of the glenoid implantation (**a**) as per the “50% rule” and previous estimation of joint line medialization (**b**) (in Reflect (Akunah, Brisbane, Australia)—for demonstration purposes only).

In addition, care must be taken when selecting the glenoid implant type and design, as every device lateralizes the baseplate–glenosphere construct to a different degree. A combination of patient factors, such as patient size and body habitus, paired with estimated joint line medialization must be taken into account (Figure 6).

##### Humerus

As discussed previously, preoperative planning for the revision of the humerus allows the surgeon to ascertain the amount of bone deficiency and employ a variety of planning tools to identify prosthesis parameters. These include version, inclination, and sizing of the primary implant. More importantly, planning enables the surgeon to gauge the size of the stem and evaluate strategies for removal of the primary implants with potential osteotomies.

Strategies must then be employed by the surgeon to sufficiently compensate for the humeral bone loss, whether using metallic augments, stem selection, specific Allograft Prosthetic Complexes (APC), or tumor prostheses.

*Bone Deficiencies Proximal to the Pectoralis Major Tendon Insertion (<6 cm)*

These can generally be compensated for by keeping the implant proud while employing cementation techniques [37]. Bone grafting of the residual tuberosities and fixation to the stem generally result in a suitable outcome. It should be noted that the humeral length still needs to be restored, and the temptation to stack multiple liners (“tower of terror”) may result in recurrent instability postoperatively.

*Bone Deficiencies below the Pectoralis Major Origin but Proximal to Deltoid Insertion (6–14 cm)*

**Figure 7:**
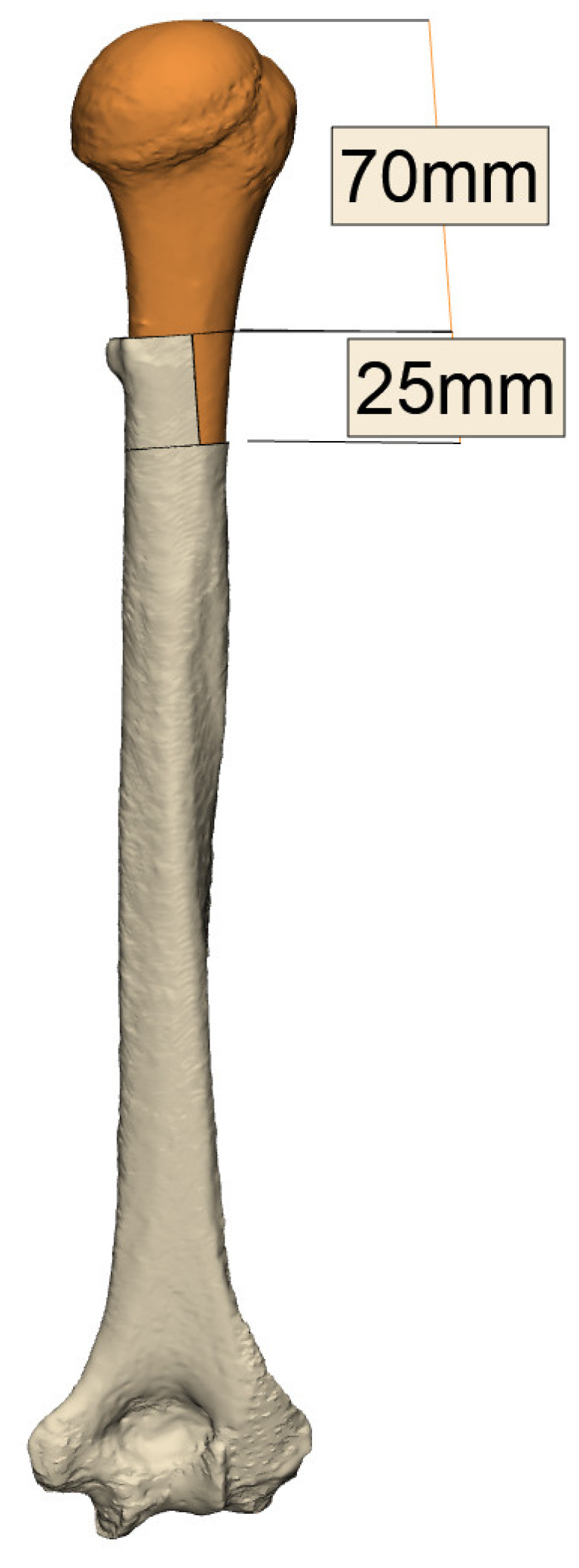
Planned APC dimensions to compensate for humeral bone loss (images from Reflect COMPLEX^TM^ (Akunah, Brisbane, Australia)).

These generally cannot be compensated for by keeping the stem proud. Accurate reconstruction of the humeral deficiency, either with an APC or proximal humeral replacement prosthesis, is advised (Figure 7) [37]. Care must be taken to preserve the deltoid insertion at all costs, as this has a significant bearing on the patient’s early postoperative active abduction and flexion.

*Bone Deficiencies Below the Deltoid Insertion (>14 cm)*

These are complex deficiencies and necessitate accurate humeral length planning/restoration and meticulous soft tissue reconstruction and reattachment of the deltoid, latissimus dorsi, pectoralis major, and the residual cuff to ensure stability as these deficiencies predispose to recurrent instability.

### 2.3. Soft Tissue Condition

In addition to the bony anatomy and the implants, the surrounding soft tissues need to be evaluated as well. The skin must be inspected for any sinus, as this confirms the presence of infection. Although this is not a contraindication for the single-stage revision, this indicates the need for thorough and more meticulous debridement and strict implementation of the postoperative antibiotic protocol.

The presence of scars and the status of the soft tissue envelope need to be assessed. The possible remaining soft tissue structures after debridement need to be predicted in order to preoperatively plan for the reconstruction that may be required to achieve good function, such as tendon transfers. If the skin and remaining soft tissue envelope are predicted to be at risk of having poor healing, or primary closure might not be possible, then single-stage revision may not be the best option.

## 3. Surgical Technique

### 3.1. Two Step Single-Stage Procedure

**Figure 8:**
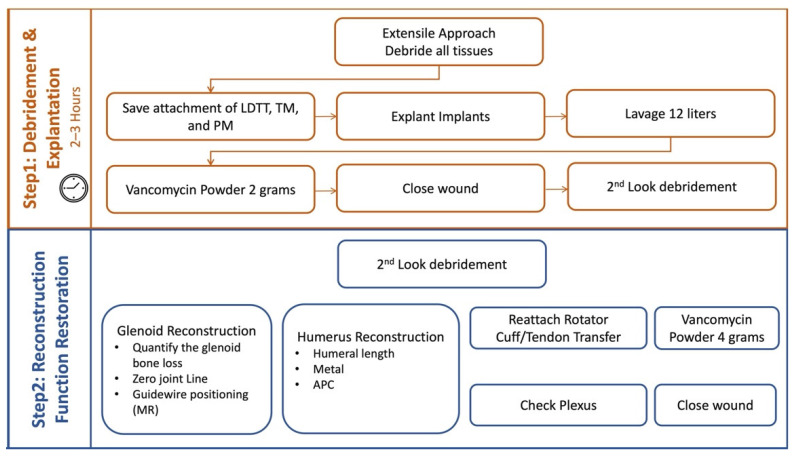
Surgical process for two-step single-stage revision shoulder arthroplasty.

The single-stage revision shoulder arthroplasty is accomplished in two steps. The first step involves debridement and explantation of implants, and the second step involves reconstruction and restoration of function (Figure 8).

*First Step*

The technique starts with an extensile approach. The deltopectoral approach is utilized as this can be extended proximally and distally as necessary. Clavicle osteotomy may be performed if a more extensile approach is necessary for better exposure of the surgical site. Any sinus or scar tissue is excised. Thorough and meticulous debridement of soft tissues is done. This is the key to a successful single-stage revision. Patience and meticulous surgical debridement are key virtues needed by all surgeons involved. All devitalized tissues are removed until healthy tissue is noted. Attention to the attachments of vital tendons is crucial, specifically latissimus dorsi, teres major, and pectoralis major tendons. These are important to be spared and tagged in order to achieve good function postoperatively.

Attention to neurovascular structures is crucial. Often in a revision, the plexus and the axillary nerve are scarred and often a source of pain. Neurolysis is usually necessary for revision surgeries. There is a high risk of neuropraxia or plexopathy postoperatively as there is a possibility for the neurovascular structures to be stretched out after reconstruction, especially with longstanding contractures and multiple past operations. Scar tissue, adhesions, and fibrotic tissues are meticulously excised. Dissection around these vital structures is challenging; therefore, careful dissection with the aid of a nerve stimulator is recommended to safely tackle this step. Loupes are routinely worn while dissecting the neurologic structures, specifically when dissecting the glenoid capsule, where excision of the inferior glenoid capsule is necessary, and the axillary nerve and its branches need to be identified and preserved.

A complete 360-degree peri-glenoid capsular excision is mandatory to achieve complete glenoid visualization. The subscapularis is peeled off the glenoid neck to visualize 3 cm of the anterior glenoid neck and the base of the coracoid. The inferior glenoid capsule is completely excised. This thick hammock of tissue is a major cause of recurrent instability.

Assessment of the glenoid implants in situ is then done. If the preoperative planning identifies an ideal positioning of the implants in situ, the feasibility of proceeding with retaining the implant is confirmed by assessing if the components are well-fixed or not. A properly positioned, well-fixed implants may then be retained. Otherwise, all implants are explanted carefully. Techniques to explant well-fixed implants are utilized, such as the use of curved saws and osteotomes. In well-fixed pegs, a glenoid neck window osteotomy described by the senior author (AG) may be needed, wherein a 1 cm x 1 cm bone window is created medial to the tip of the peg along with sequential disimpaction of the undersurface of the baseplate. This allows for successful removal of a well-fixed implant with minimal bone loss. Care must be taken to preserve bone stock, and any attempts to lever the baseplate out must be resisted.

Well-fixed humeral implants may be removed with a variety of techniques involving Kirschner wires, osteotomes, drill router bits, etc. Care must be taken to preserve the insertion of the cuff, pectoralis major, latissimus dorsi, and teres major. If there is poor bone stock and proximal humerus replacement is contemplated, then all these soft tissue structures must be reflected along with their osseous bony insertions to allow for bone-to-bone healing after reconstruction.

Finally, a second look debridement is performed. This is followed by lavage to thoroughly wash out the joint. At least twelve liters of normal saline is used for this step. At the conclusion of the first step, the wound should be clean, all bones viable with significant punctate bleeding, all soft tissues and muscle–tendon units tagged, and the nerves should be free. Then, 2 grams of vancomycin powder is applied, and skin is closed with 1.0 nylon suture. This first half of the technique usually takes 2 to 3 hours to complete.

*Second Step*

The second step starts. All used instruments are replaced with new ones, the patient is re-draped, and the surgical team re-scrubs as well. Second look debridement is done first. Washout and removal of residual devitalized tissues are repeated. Glenoid reconstruction then commences. The glenoid deformity that results from the removal of implants is assessed. The amount of glenoid bone loss and the remaining glenoid bone stock are quantified and compared with the holograms created from the preoperative planning. Recreation of the glenoid joint line and implantation of the glenoid components are then executed based on preoperative planning. Bone grafting of the glenoid using a wedge or Figure 7 allograft is done in the majority of cases to restore the glenoid joint line. Proper implant positioning is a challenge, especially in these cases where anatomy is significantly altered. MR is utilized in this step as guidance to achieve accurate glenoid guidewire positioning. Implantation of remaining glenoid components subsequently follows. The “50% rule” for glenoid baseplate stability is strictly followed in every case. Often in advanced glenoid defects, the alternate centerline proposed by Frankle et al. is employed as per the preoperative planning [38].

Reconstruction of the humerus then follows. The amount of remaining bone stock is assessed and confirmed with the preoperative planning. Planned reconstruction to recreate humeral length is then performed. If significant soft tissue contractures are present and/or a resection arthroplasty has been performed prior, it is a recommendation from the senior author (AG) to generally undersize the APC and/or prosthesis by 15–20% from the true humeral length preoperatively planned. A perfect size-to-size match in these conditions does not allow for reduction of the construct, and often the residual joint is very tight, which can lead to excessive plexus stretch. This shortening is compensated for by the distalization of around 3 cm in RSAs afforded by the glenosphere and the humeral cut, especially in a 155-degree design.

### 3.2. Execution with Mixed Reality (MR)

**Figure 9:**
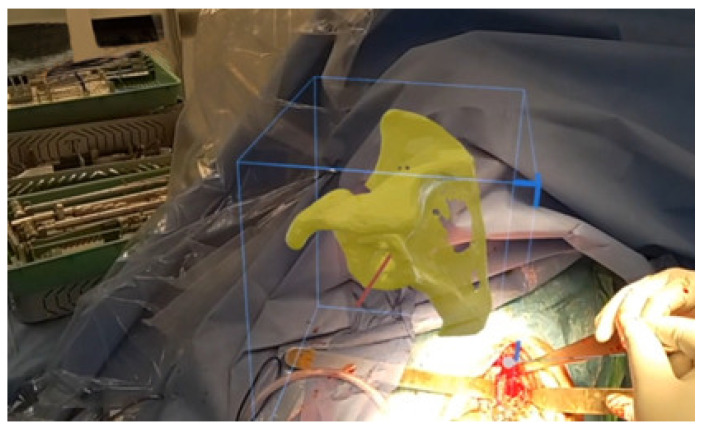
Intraoperative use of a hologram for surgical execution.

Despite advances in preoperative planning techniques, a gap still exists between preoperative planning and surgical execution. MR technology for surgical visualization aims to bridge this gap. Guidewire positioning in a revision situation is particularly challenging. The ability to visualize the glenoid deformity in 3D and manipulate in real-time the preoperative plan with 3D holograms significantly improves anatomical understanding and empowers surgical confidence (Figure 9).

In addition, in revision situations, it is common for surgeons to employ different plans and options to tackle potential intraoperative challenges. MR technology provides the advantage of generating multiple holograms from the revision preoperative plan and the various backup plans which have been established to account for varying different surgical scenarios. This is a strategic advantage over standard Patient-Specific Instrumentations (PSI), which usually only produce a single jig for a specific plan and cannot account for fallback options.

### 3.3. Soft Tissue Balancing and Reconstruction

In revision shoulder arthroplasty, one of the most critical and key features of preventing instability postoperatively is an adequate reconstruction of the soft tissue envelope, allowing for a stable construct and promoting satisfactory functional outcomes. This requires the preservation of the deltoid and as much of the rotator cuff as possible. Whenever possible, remaining rotator cuff tendons are reattached. However, cuff deficiencies are often encountered in revision settings with poor preoperative function. Restoration of this function is of paramount importance to provide stability to the implant and improve postoperative function and range of motion. This can be achieved with the employment of tendon transfers, such as latissimus dorsi tendon transfer in cases where the infraspinatus cannot be reattached and with poor preoperative external rotation. Lower trapezius transfer has been employed by the senior author (AG) in recalcitrant cases where the latissimus dorsi cannot be salvaged.

Prior to implantation of the humeral components, the proximal humerus is prepared for the reattachment of remaining rotator cuff tendons, or tendons to be transferred as necessary. All-suture anchors (2.9 mm Juggerknot^TM^ Zimmer Biomet, Warsaw, Indiana) are employed to allow for stem passage in the intramedullary canal and firm fixation of the tendons. Once this is done, humeral components are implanted and the joint is reduced. Stability is assessed. Adequate tension is checked by assessing the tension of the soft tissues, such as the conjoint tendon, which is a key stabilizer of the shoulder girdle. In addition, the tension on the axillary nerve, radial nerve, and brachial plexus is assessed. Rotator cuff tendon reattachment or tendon transfers are then performed. Lavage with peroxide diluted with saline and a second mix of betadine and saline is done throughout the case, followed by application of 4 grams vancomycin powder and wound closure.

### 3.4. Postoperative Antibiotic Regimen 

**Figure 10:**
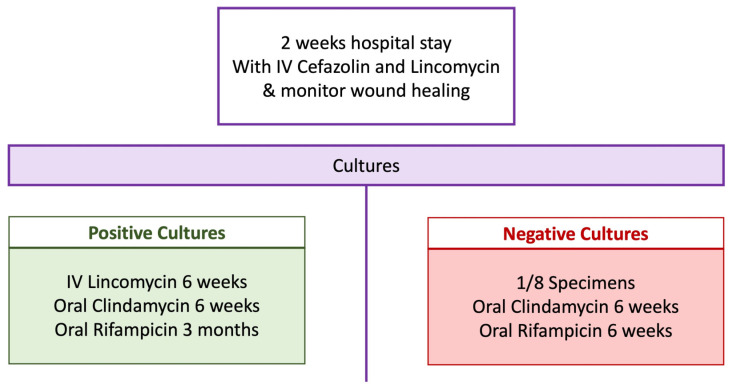
Postoperative antibiotic regimen.

Aside from the local antibiotic delivery achieved by the administration of vancomycin powder intraoperatively, a systemic postoperative antibiotic regimen (Figure 10) is crucial to the success of single-stage revision. The patient is confined to the hospital for two weeks for initial intravenous (IV) antibiotic therapy and for monitoring wound healing. The choice of antibiotics for this period is cefazolin and lincomycin. For those who have positive intraoperative cultures, 6 weeks of IV lincomycin is given, followed by 6 weeks of oral clindamycin and 3 months of rifampicin. For those who have negative intraoperative cultures, oral clindamycin for 6 weeks and oral rifampicin for 6 weeks are given. It must be noted that the antibiotic regimen proposed is as recommended by an infectious disease specialist based on the local sensitivities. Local factors and sensitivities must be checked regionally. Otherwise, culture-guided antibiotic treatment can be done. Moreover, the addition of rifampicin to the standard-of-care for PJI has been shown to confer a protective effect against treatment failure following PJI, which can be attributed to its ability to penetrate biofilms [39].

## 4. Preliminary Results

Retrospective review of our institutional surgical database identified 31 revisions of shoulder arthroplasty in 30 patients (mean age 72 ± 7 years old, 15 males and 15 females) performed by the senior author between June 2016 and December 2021. Informed voluntary consent was given by all participants, and ethics approval was obtained (Ramsay Health Care QLD HREC Protocol ID 20/23).

There were 27 revisions of Reverse Shoulder Arthroplasty (RSA), 2 revisions of Total Shoulder Arthroplasty (TSA), and 2 failed complex Open Reduction Internal Fixations (ORIF). The majority of the revisions were complex procedures requiring significant glenoid and/or humeral allografts and tendon transfers to compensate for soft tissue loss. No custom implants were used in our series.

Out of 31 revisions, 24 had a single-stage procedure (mean age 71 ± 7 years). Etiologies for single-stage revision are presented in Table 2.

All revisions that were preoperatively planned were successfully executed. The 3D-reconstructed models prior to and after implant subtraction provided as part of preoperative planning activities were found to accurately match intraoperative findings. The surgeon was able to successfully use the pre-planned glenoid guidewire position and humeral dimensions intraoperatively in all cases.

Patient follow-up is still ongoing, but preliminary results on functional improvements at a mean of 1 year follow-up for single-stage revisions are displayed in Table 3 [40].

Most patients who underwent a revision came from multiple different facilities due to the tertiary nature of care provided and needed to be admitted to the rehabilitation facility for convalescence; thus, the inpatient stay of an extra two weeks is noted in our results for some patients.

In addition, all infections were successfully addressed and treated with debridement, implant revision or retention, and postoperative IV antibiotics. Single-stage revisions were successful in treating frank infections. The time to normalization of serological markers was 2 weeks. *Cutibacterium acnes* and *Staphylococcus aureus* were the two dominant organisms cultured. There has been no recurrence of infection in our cohort of 31 patients. There has been no instability postoperatively. Three patients had postoperative neural symptoms, which resolved within 6 months. One patient had persistent ulnar nerve neuropraxia, which existed preoperatively.

Finally, preliminary costs analysis and estimations showed that, on average, a single-stage stage revision allowed for a two-thirds reduction in hospital costs, an average saving of AUD $210,000 per hospital stay in our facility [40]. This further highlights the importance of preoperative planning to assist in saving costs to the healthcare service, the patient, and the surgeon. A two-step single-stage procedure has enabled a reduction in the postoperative care to 3 months rather than a staged procedure whereby the patient may not be able to return to work for 6 to 9 months, thereby providing a cost saving of roughly $100,000 in income and wages loss for each patient.

## 5. Discussion

3D preoperative planning with metal artifact reduction for revision shoulder arthroplasty offers significant advantages to the surgeon, the patient, and the healthcare service.

Preoperative planning with an agnostic software is beneficial for the surgeon as it provides significant clinical information regarding the anatomy and allows us to analyze the primary implants in situ. This will further guide the surgeon on the strategies to adopt for the revision procedure, such as whether to retain the implant in situ or to completely remove primary implants and proceed with re-implantation. Subtraction of the primary implants assists the surgeon in understanding existing bony deficiencies and achieving accurate reconstruction. It also allows for multiple different plans to be created, thus simulating different scenarios and evaluating multiple strategies based on the availability of implants. These factors allow for single-stage revision procedures to be undertaken and improve the surgeons’ and their teams’ confidence when dealing with complex revision arthroplasty cases.

The patient benefits from these as single-stage procedures reduce the number of operations, thus reducing patient morbidity and length of hospital stay while achieving satisfactory postoperative outcomes.

Finally, preoperative planning also benefits the healthcare system by reducing the costs involved with revision procedures. In addition, allowing the surgeon to carefully evaluate reconstructive implants required assists in reducing the inventory requirements for revision surgery by reducing the number of trays that need to be sterilized [41] and further minimizes the logistic support required from the implant manufacturers.

## 6. Conclusions

To conclude, this article presented a detailed plan and strategies for the management of revision of shoulder arthroplasty. We have shown that these can be successfully managed with a two-step single-stage procedure when paired with preoperative planning. The use of MR technology also presents tremendous potential to bridge the gap between planning and surgical execution.

## Figures and Tables

**Table 1:** Factors to take into consideration for the management of revision shoulder arthroplasty.

Patient Factors	Bony Anatomy	Implants	Soft Tissue Balancing
Age Comorbidities Fragility of the patient Bone quality Presence of infection Presence of neurogenic pain	**Glenoid** ○ Bone loss ○ Residual bone stock for reconstruction ○ Joint line	**Existing implants** ○ Current positioning ▪ Correct ▪ Malpositioned ○ Baseplate and humeral stem fixation ▪ Stable ▪ Unstable ○ Extended glenosphere	Excision of scar tissue Axillary neurolysis Cuff reattachment Pectoralis major, teres major, and deltoid attachment Tendon transfers
	**Humerus** ○ Bone loss ○ Residual bone stock for reconstruction ○ Humeral length	**Revision implants** ○ Fixation options ▪ Long peg ▪ Screw ○ Augments ○ Humeral stems ▪ Design ▪ Sizing ○ Allografts ○ Tumor implants ○ Custom implants	

**Table 2:** Etiology for single-stage revision procedures.

Etiology	
Infection	17%
Pain	21%
Implant loosening	8%
Fracture	8%
Other	21%

**Table 3:** Preliminary results on functional outcomes following a single-stage revision procedure at mean 1-year follow-up.

	Preop	Postop
Total Hospital stay (days)	16 ± 13	
Forward Flexion (deg)	65 ± 52	142 ± 29
Lateral Elevation (deg)	58 ± 43	128 ± 36
External Rotation (deg)	14 ± 23	38 ± 22
Patient satisfaction (%)	5	87

## Data Availability

Not applicable.
